# Increasing the Permeability of Polyphenylene Sulfone Hollow Fiber Ultrafiltration Membranes by Switching the Polymer End Groups

**DOI:** 10.3390/polym17010053

**Published:** 2024-12-29

**Authors:** Alisa Raeva, Dmitry Matveev, Tatyana Anokhina, Azamat A. Zhansitov, Svetlana Khashirova, Vladimir Volkov, Ilya Borisov

**Affiliations:** 1 Center for Progressive Materials and Additive Technologies, Kabardino-Balkarian State University Named After H.M. Berbekov, 360004 Nalchik, Russia; azamat-z@mail.ru (A.A.Z.); new_kompozit@mail.ru (S.K.); vvvolkov@ips.ac.ru (V.V.); 2 Laboratory of Polymeric Membranes, A.V. Topchiev Institute of Petrochemical Synthesis Russian Academy of Sciences, 119991 Moscow, Russia; dmatveev@ips.ac.ru (D.M.); tsanokhina@ips.ac.ru (T.A.)

**Keywords:** polyphenylene sulfone, end groups, hollow fiber membrane, ultrafiltration, high permeance

## Abstract

The influence of the molecular weight and chemical structure of polyphenylene sulfone (PPSU) end groups on the formation of the porous structure of ultrafiltration (UF) hollow fiber membranes was investigated. Polymers with a molecular weight ranging from 67 to 81 kg/mol and with a hydroxyl-to-chlorine end group ratio ranging from 0.43 to 17.0 were synthesized. The excess of end groups was achieved during polymer synthesis by adding one of the following monomers: hydroxyl (excess DHBP) or chlorine (excess DCDPS). For the first time, it was found that the stability of PPSU solutions is determined not by the molecular weight of the polymer, but by the chemical structure of its end groups. The stability of polymer solutions increases with the increasing proportion of chlorine groups. The SEM method showed that with the increasing molar fraction of chlorine end groups in the polymer, a more open porous structure forms on the outer surface of the hollow fiber membranes derived from it. The maximum UF permeance of the hollow fiber membranes for water was achieved with the PPSU sample containing the highest chlorine end group content, amounting to 136 L/(m^2^·h·bar), with a high rejection of the model substance Blue Dextran (at 94.7%). This represents the best result currently reported among unmodified PPSU hollow fiber membranes.

## 1. Introduction

Free access to clean drinking water is one of the fundamental factors in the preservation of human health. At the same time, only 1% of the planet’s abundant water resources are suitable for use as drinking water, even after treatment [1]. In recent decades, declining water quality, which is primarily caused by human activities, has increasingly become a problem. According to the WHO, in 2022, at least 1.7 billion people used water contaminated with toxic substances and various microorganisms [2]. For this reason, modern and reliable methods for industrial and domestic wastewater treatments are needed.

Baromembrane separation processes, namely microfiltration and ultrafiltration processes, are actively gaining popularity among the processes of removing various colloids and organic molecules from water [3]. This aspect is related to the clear advantages of membrane technology over traditional water treatment methods: compactness, cost-effectiveness, and stability.

The global membrane filtration market was worth over USD 16 billion in 2023 and is projected to grow steadily due to increasing incidences of water contamination and waterborne diseases [4]. It has been observed that microfiltration membranes are the most effective in bacteria removal, while in cases of virus removal, ultrafiltration membranes are the most effective [5,6]. It should be noted that researchers and industrialists are more interested in the hollow fiber configuration of membranes. Due to its comparative compactness, such a module can accommodate the largest surface area for contact with the mixture to be separated [7].

Polymeric materials currently dominate the membrane market due to their low cost as well as ease of production and scalability. Polysulfone, polyethersulfone, polyether sulfone, polytetrafluoroethylene, polyamides, cellulose acetate, etc., are actively used to produce membranes [5,8,9]. However, in the filtration of media containing pathogenic organisms, additional requirements are imposed on membranes, primarily sterilization ability [10].

Among the wide range of polymeric membrane materials (e.g., polytetrafluoroethylene (PTFE), polysulfone (PSF), and polyethersulfone (PES)), polyamides are mechanically and hydrolytically resistant to the effect of superheated steam [11,12,13,14]. The disadvantage of PTFE is that it is expensive during production, and on this basis, it is possible to obtain only the microfiltration membrane via sintering since this polymer is insoluble in organic solvents [15]. Polyamides are used to produce membranes that can withstand several steam treatment cycles, but the abilities of this polymer are strictly limited [16]. The polymers of the polyarylsulfone series—PSF and PES—are of the greatest interest in this topic. They currently occupy a large share of the ultrafiltration membrane market. These polymers have high chemical stability and are known for their superior performance and high retention capacity compared to other polymeric materials. However, they are also prone to degradation during steam treatments, withstanding no more than 100 cycles [17].

Another polymer in the polyarylsulfone range is polyphenylene sulfone (PPSU). According to the data presented by BASF, this material can withstand superheated steam treatments without losing its original properties for up to 2000 treatment cycles [17]. In this regard, it appears to be an attractive alternative to existing membrane filters subjected to similar operations.

From the literature, it is known that PPSU has been used as a membrane-forming material in various filtration processes, such as ultrafiltration of aqueous media [18,19,20,21,22,23,24], nanofiltration of organic media [22,25,26], pervaporation of acetone/water, alcohol/water mixtures [27,28], the separation of butanol from acetone–butanol–ethanol mixtures [29], as well as gas separation [30,31]. It has been shown that ultrafiltration membranes from PPSU can be used for water purification, water treatment [18,20,23], metal removal [21,24], protein separation [18], and oil/water mixtures [32].

One of the disadvantages of PPSU as a membrane material is its hydrophobicity, which increases its tendency to foul [33]. For this reason, researchers often resort to modifying the original PPSU in order to increase the hydrophilicity of the material [34]. However, such modifications tend to have a negative effect on the performance characteristics of PPSU. It should be noted that the mechanical strength of homopolymer PPSU ensures its high stability during sterilization and regeneration with superheated steam. The literature shows that the best steam treatment effect is achieved if the module sterilization is carried out directly in the technological filtration line [35]. Such a design allows for minimizing the risk of pathogens entering the sterile environment of the unit and their further multiplication. For this reason, the development of membranes made of unmodified PPSU is an urgent task.

The literature presents works on the production of hollow fiber membranes from PPSU. As we have shown earlier [36], for the most part, commercial grades of PPSU with a molecular weight of 48–65 kg/mol are used to obtain membranes. The best result in the filtration experiment for flat-sheet unmodified membranes was obtained using PPSU with M_w_ = 48 kg/mol (polymer grade Ultrason P 3010 NAT, BASF)—650 L/(m^2^·h·bar); the rejection of human serum albumin (HSA) for such a membrane was 83% [37]. It should be noted that the temperature of the coagulation bath used to obtain this membrane was 70 °C. In [38,39], the results for the production of flat-sheet membranes from PPSU with M_w_ = 78 kg/mol are presented. The water permeance values of such membranes were 450 L/(m^2^·h·bar) [39] and 848 L/(m^2^·h·bar) [38]. The authors noted that the membranes were stored in 1% formaldehyde solution, which probably could have influenced the filtration properties of the presented membranes.

It should be noted that it is difficult to obtain hollow fiber membranes based on commercial grades of PPSU due to the insufficient molecular weight of polymers. Previously, a hollow fiber membrane obtained from synthesized PPSU with a molecular weight of M_w_ = 102 kg/mol was developed [36]. The permeance of such a membrane was 95.7 L/(m^2^·h·bar) with a rejection of the model substance Blue Dextran (M_w_ = 70 kg/mol) of 99.9%. Currently, this is the best result for the hollow fiber membrane obtained from the unmodified PPSU. It should be emphasized that the scientific literature does not contain any works devoted to the development of polyphenylene sulfone hollow fiber membranes with a molecular weight in the range of 48–65 (from commercially available PPSU) to 102 kg/mol (synthesized in laboratory conditions). Moreover, the literature lacks studies on the influence of the chemical structures of the end groups on the filtration properties of the final product.

Therefore, the following objectives were set in the present work: to synthesize PPSUs in a narrower range of molecular weights featuring different contents of end hydroxyl and chlorine group; to investigate the characteristics of the synthesized polymers; to prepare and investigate the properties of dope solutions; to prepare ultrafiltration hollow fiber membranes and investigate their morphology, transport, and separation properties.

## 2. Materials and Methods

### 2.1. Materials

For the synthesis of PPSU, 4,4′-dihydroxybiphenyl (DHBP, Alfa Aesar, Morecambe, UK) and 4,4′-dichlorodiphenylsulfone (DCDPS, Alfa Aesar, Morecambe, UK) were used as monomers, N,N-dimethylacetamide (DMAc, Acros Organics, Geel, Belgium) was used as the solvent, and potassium carbonate (Reachem, Moscow, Russia) was used as the alkaline agent.

N-methyl-2-pyrrolidone (NMP, Chimmed, Moscow, Russia) was used as a solvent in the preparation of polymer solutions. Polyethylene glycol (PEG) with a molecular weight of 400 g/mol (Sigma-Aldrich, St. Louis, MO, USA) was used as a pore-forming additive.

An aqueous solution of the model substance Blue Dextran (Sigma-Aldrich, USA) with a molecular weight of 70 kg/mol was used to study the transport and separation properties of hollow fiber membranes.

### 2.2. Synthesis of PPSU

The synthesis of PPSUs was carried out in a four-neck reaction vessel equipped with a thermocouple, a capillary for inert gas supply, a mechanical stirrer, a Dean-Stark trap, and a reflux condenser. For the non-equilibrium polycondensation reaction, DHBP and DCDPS were loaded into the vessel at the ratios given in Table 1.

The reaction mixture was heated to the boiling point of DMAc (165 °C) by stirring under inert gas (nitrogen). Solvent distillation was stopped when the mixture reached the boiling point of the solvent. The time of the synthesis was 4 h. After that, the temperature of the mixture was reduced to 90 °C by adding a solution of oxalic acid in DMAc. Then, the reaction mixture was purified from the formed salts and PPSU was precipitated into distilled water by spraying. After that, the polymer was washed with water and dried at 150 °C in a drying chamber [40].

### 2.3. Characterization of Synthesized PPSUs

#### 2.3.1. Gel Permeation Chromatography (GPC)

The molecular weight characteristics of the synthesized PPSUs were investigated using a Waters refractometer detector (Chromatopak Microgel-5). Polymer solutions were prepared in chloroform at a concentration of 1 mg/mL. The characteristics of PPSU were calculated with respect to monodisperse polystyrene standards [36].

#### 2.3.2. Nuclear Magnetic Resonance (NMR)

^1^H NMR was performed according to the standard procedure using a Bruker AVANCE III HD 400 NMR spectrometer. Deuterated chloroform was used as a solvent for the synthesized PPSUs. The average molecular weight (M_NMR_) for each synthesized sample was calculated as the ratio of the area of the peak corresponding to the segment of the main polymer chain to the areas of the peaks corresponding to the signals of the end hydroxyl and chlorine groups.

#### 2.3.3. Differential Scanning Calorimetry (DSC)

DSC analysis was performed on a Perkin Elmer DSC 4000 instrument in an inert medium in the temperature range of 30–250 °C. The scanning speed was 10 C·min^−1^. The glass transition temperature values obtained during the second heating of the sample were taken as the results of the analysis.

#### 2.3.4. Determination of Coagulation Values

The stability of PPSU solutions in NMP was investigated by measuring the coagulation values (CV, g/dL). This value is characterized as the amount of coagulant (1 g) required to phase-disintegrate 100 mL (1 dL) of polymer solution. The study was carried out via the titration method; distilled water was used as a titrant. For this purpose, 2 wt.% solutions of PPSUs in NMP were prepared. The deposition process was observed visually.

### 2.4. Preparation of PPSU Solutions and Measurement of Viscosity

As part of the work, solutions based on the synthesized PPSUs were prepared with the composition shown in Table 2.

The dynamic viscosity of solutions was determined using an Anton Paar MCR 72 rotary rheometer (Anton Paar, Graz, Austria) equipped with a CP60-0.5 cone-plane measuring unit. The shear rate was 10 1/s, and the temperature was fixed at 23 °C.

### 2.5. Preparation of Hollow Fiber PPSU Membranes

During membrane doping, solutions of the following composition were used: PPSU/NMP/PEG = 20/50/30 wt.%. The hollow fiber membranes were obtained using the dry-jet wet method (the “free spinning” variant), where the formed hollow fiber under the action of gravity entered the receiving bath with water. Distilled water was used to form the inner channel. A ring spinneret with outer and inner diameters of 0.8 and 0.5 mm, respectively, was used. After doping, the samples of hollow fiber membranes were washed in distilled water for at least 17 h.

### 2.6. Measurements of Transport and Separation Properties of Hollow Fiber PPSU Membranes

The study of transport and separation characteristics was carried out in the setup described in [41]. Measurements were carried out via a cross-flow mode with a transmembrane pressure of 1 bar. The permeance was calculated according to Formula (1):

(1) $$P=\frac{V}{S\cdot t\cdot ∆p},$$

where *P*—permeance, L/(m^2^·h·bar); *S*—surface area of the selective layer of the hollow fiber membrane, m^2^; *V*—volume of the selected sample, L; *t*—sampling time, h; Δ*p*—overpressure, bar [41]. 

To investigate the rejection, an aqueous solution of the model substance Blue Dextran, with a concentration of 100 mg/kg, was prepared. The rejection was calculated according to Formula (2):

(2) $$R=\frac{1-C_{perm}}{C_{feed}}\cdot 100\%,$$

where *R*—rejection, %; *C_perm_*—concentration of the dissolved substance in the permeate, (mg/L); *C_feed_*—concentration of the dissolved substance in the initial flow, mg/L [41].

### 2.7. Scanning Electron Microscopy (SEM)

The SEM method was used to characterize the porous structure of hollow fiber membranes from PPSU. A Thermo Fisher Phenom XL G2 Desktop SEM instrument (Thermo Fisher Scientific, Waltham, MA, USA) was used for imaging. Cross-sections of hollow fiber membranes were obtained by breaking them in liquid nitrogen media. Silver layers (5–10 nm) were deposited on the samples in a vacuum environment using a magnetron sputter coater, “Cressington 108 auto Sputter Coater” (Grassendale, Liverpool, UK) [41].

## 3. Results

### 3.1. Chemical Structure of the Synthesized PPSUs

**Figure 1:**
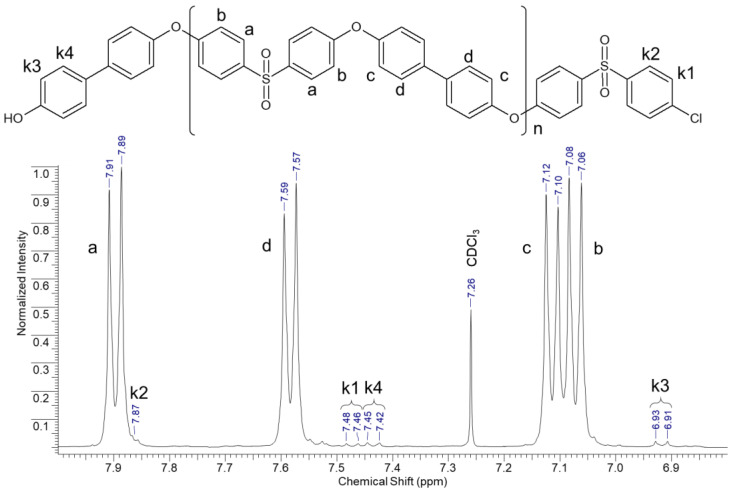
Chemical structure of polyphenylene sulfone and general view of ^1^H NMR spectrum.

The chemical structure of the polymers synthesized in this work was investigated using NMR spectroscopy. The general view of the proton spectrum is shown in Figure 1. The proton signals of the synthesized PPSUs are in the chemical shift range of 6.8–8.0 ppm.

Signals at chemical shift values δ7.90 H-a (4H, d), δ7.58 H-d (4H, d), δ7.11 H-c (4H, d), and δ7.07 H-b (4H, d) were assigned to protons of the aromatic rings of the main polymer chain. The peaks k1 δ7.47 (2H, d) and k2 δ7.87 (2H, d) were assigned to protons of the end aromatic rings connected to Cl-end groups, and the proton signals k3 δ6.92 (2H, d) and k4 δ7.44 (2H, d) were assigned to protons of the aromatic rings connected to OH-end groups. Thus, the chemical structure of the synthesized polymers as polyphenylene sulfones was confirmed. Additionally, from the obtained ^1^H-spectra, the mole fractions of hydroxyl y_-OH_ and chlorine y_-Cl_ groups were calculated (the data are given in Table 3). It is evident from the above data that the excess DHBP monomer during the synthesis of PPSU led to the predominance of end hydroxyl groups in the final polymer (samples PPSU-3 and PPSU-4). When the monomer DCDPS was used in excess during the synthesis, a natural predominance of end chlorine groups in the sample PPSU-1 was observed. At the same time, in the case of the synthesis of PPSU at the equimolar ratio of monomers (sample PPSU-2), a predominance of end hydroxyl groups in the polymer was observed.

The molecular weight characteristics of the polymers were also investigated via the GPC method. The results of the study are shown in Table 3, where it can be seen that the weight-average molecular weight, M_w_, of the synthesized PPSUs is in the range of 67–81 kg/mol. Additionally, the calculated values of the number-average molecular weights of M_n_ obtained from the NMR (M_NMR_) spectra are summarized in Table 3. It should be noted that the M_NMR_ values differ from the values of the mean number-average molecular weights of M_n_ obtained by the GPC method by a factor of 1.9–2.8. This phenomenon arises due to the relatively large molecular weight of the presented PPSUs, which reduces the resolution of the spectra and, consequently, the accuracy of the calculation [42].

### 3.2. Glass Transition Temperature of PPSUs

The glass transition temperatures (T_g_) of the synthesized PPSUs were determined using the DSC method, as presented in Table 4. Table 4 shows that the synthesized PPSUs have similar values to the glass transition temperature (T_g_—225.9–228.5 °C). The obtained data are in agreement with the results of Ref. [40], where the thermal properties of the synthesized PPSUs were presented. It was shown that the PPSUs with a molecular weight (M_w_) of 62, as well as 102 kg/mol, have glass transition temperatures (T_g_) of 220 and 227 °C, respectively.

### 3.3. Coagulation Values

**Figure 2:**
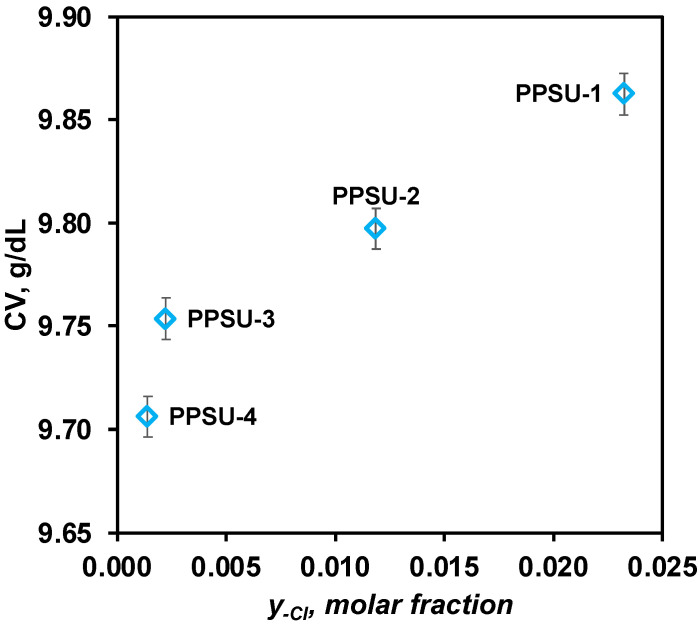
Dependence of CVs on the molar fraction of end chlorine groups in PPSUs.

In order to investigate the stability of PPSU solutions in NMP, the dependence of CV on the fraction of end chlorine groups in PPSU was found (Figure 2). It is shown that an increase in the molar fraction of the Cl-end group y_Cl_ in the structure of PPSU from 0.001 to 0.012 leads to a slight increase in CV from 9.71 to 9.86 g/dL. It follows from the above data that the amount of water required for coagulation increases as the molar fraction of the end chlorine groups increases, indicating an increase in the solubility of polymers in NMP. 

### 3.4. Properties of Dope Solutions

**Figure 3:**
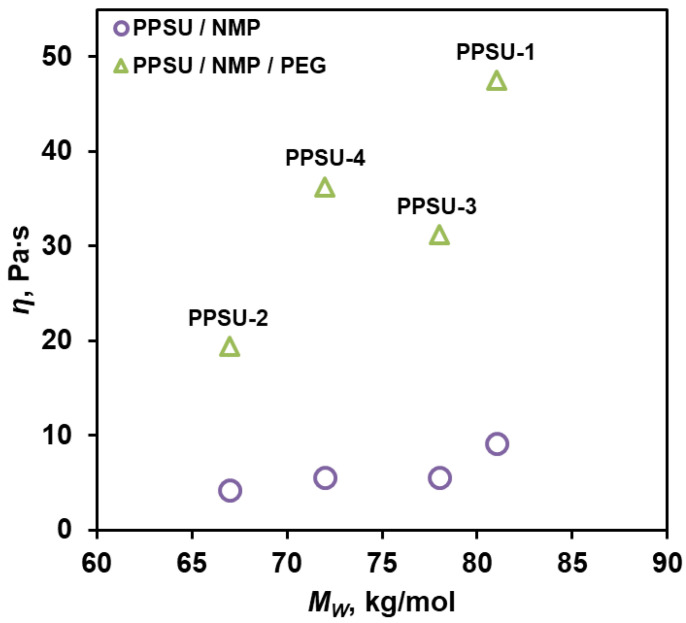
Dependence of the dynamic viscosity of two- and three-component dope PPSU solutions on the molecular weight of PPSUs.

The dynamic viscosity of the polymer solution is one of the key factors in the preparation of hollow fiber membranes. For this reason, the viscosity characteristics of solutions based on synthesized PPSUs were analyzed. For this purpose, two- and three-component solutions of the following compositions were prepared: PPSU/NMP = 20/80 wt.% and PPSU/NMP/PEG = 20/50/30 wt.%. The introduction of a third component (PEG) into the system was necessary to increase the porosity of the future membranes. The choice of the dope composition is due to the results obtained by us earlier and presented in [36]. The measurement results are shown in Figure 3.

Figure 3 shows that as the molecular weight of polymers increases from 67 to 81 kg/mol, the viscosity of solutions (in the case of the PPSU/NMP system) increases from 4.3 to 9.2 Pa·s. It should be noted that PEG further increases this parameter, so the viscosity of solutions in the case of the PPSU/NMP/PEG system ranges from 19.4 to 47.5 Pa·s as the molecular weight of PPSU increases from 67 to 81 kg/mol.

It should be noted that for polymers PPSU-4 and PPSU-3, an inverse dependence of the dynamic viscosity of the solution on the molecular weight of the polymer was observed. Moreover, when PEG was introduced, this aspect was more noticeable. This phenomenon was likely caused by the varying polydispersity of the synthesized samples. According to the GPC data (Table 2), the polydispersity coefficients for PPSU-4 and PPSU-3 samples were 1.6 and 2.4, respectively. It is known that the weight-average molecular weight, M_W_, describes the contribution of the high molecular weight fraction in the polymer, while the number-average molecular weight, M_n_, describes the contribution of the low molecular weight fraction. It follows that the higher the polydispersity coefficient, the greater the contribution of the low molecular weight fraction to the dynamic viscosity, underestimating this value.

### 3.5. Characterization of Hollow Fiber Membranes from Synthesized PPSUs

**Figure 4:**
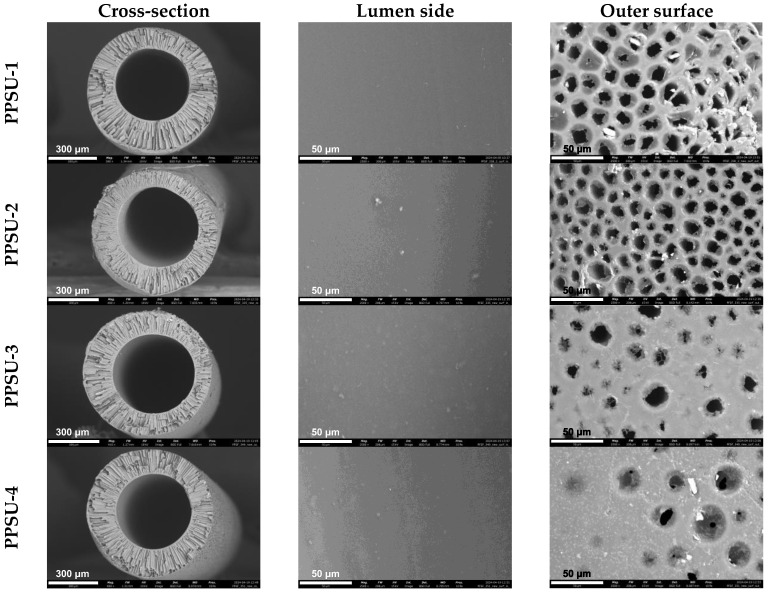
SEM images of hollow fiber membranes from synthesized PPSUs.

At the next stage of the work, hollow fiber membranes were obtained from synthesized PPSUs from dope solutions of composition PPSU/NMP/PEG = 20/50/30 wt.%. The SEM images of the obtained membranes are shown in Figure 4, where it can be seen that all the obtained hollow fiber PPSU membrane samples possess a developed finger-like porous structure in the support layer. It is also evident from Figure 4 that all the submitted hollow fiber PPSU membranes possess a dense layer on the lumen side. It should be noted that as the molar fraction of the end chlorine group y_Cl_ increases from 0.001 to 0.012, the number of finger-like pores extending to the outer surface of the hollow fiber membranes increases in the series of PPSU-4, PPSU-3, PPSU-2, and PPSU-1. The obtained porous structures of hollow fiber PPSU membranes agree well with the data obtained during the filtration experiment.

**Figure 5:**
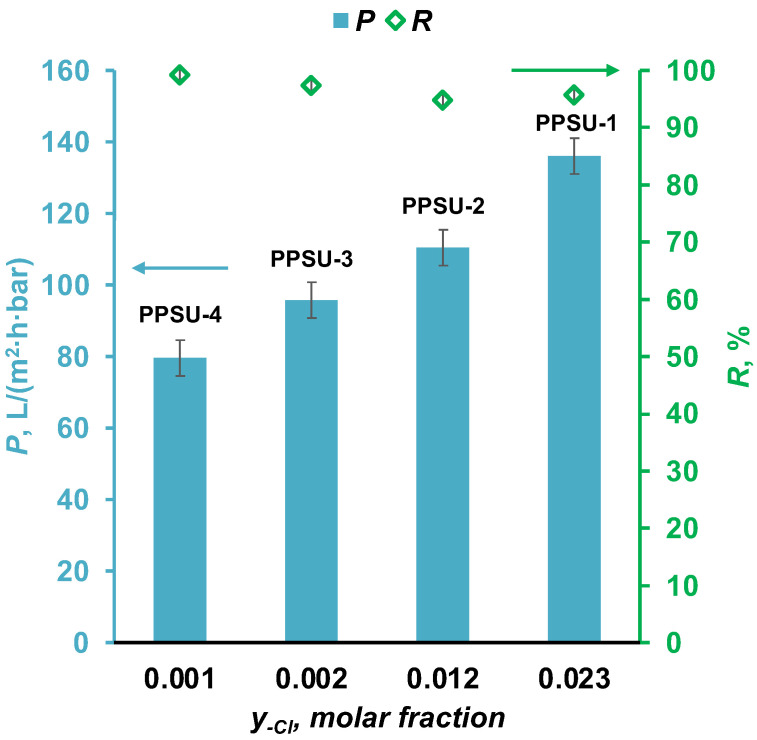
Dependence of transport and separation properties of hollow fiber PPSU membranes on the molar fraction of end chlorine groups.

The results of the study of transport and separation properties are shown in Figure 5. It is shown that when the molar fraction of the end chlorine group y_Cl_ increased from 0.001 to 0.023, the permeance of the hollow fiber membranes increased significantly from 79 to 136 L/(m^2^·h·bar). Meanwhile, the rejection rates of the presented hollow fiber PPSU membranes, investigated using an aqueous solution of the model substance Blue Dextran with a molecular weight of M_w_ = 70 kg/mol, ranged from 94.7 to 99.2%.

It can be concluded that at a relatively close molecular weight of the synthesized PPSUs (67–81 kg/mol), the permeance of the hollow fiber membranes was more influenced by the chemical structure of the end groups. Thus, in this work, for the first time, an ultrafiltration membrane made of unmodified PPSU with a molecular weight of M_w_ = 81 kg/mol, in which end chlorine groups predominated, was obtained, demonstrating water permeance of no less than 136 L/(m^2^·h·bar) and rejection of the model substance Blue Dextran of 96%.

The hollow fiber membranes obtained from synthesized PPSUs have competitive filtration characteristics in comparison with membranes (the data on which were published earlier (Table 5)). For example, the membrane obtained in this work from the solution of PPSU-1/NMP/PEG = 20/50/30 wt.% has better permeance by more than two times compared to the hollow fiber membrane obtained from commercial PPSU produced by Solvay, USA, (solution of PPSU/NMP = 20/80 wt.%)—136 L/(m^2^·h·bar) and 65 L/(m^2^·h·bar), respectively [25]. At the same time, the membranes exhibit close values of rejection for the model—95.2 and 96%, respectively [20]. It should be noted that the permeance value for the membrane in this work was calculated as an average value after the membrane reached steady-state conditions (i.e., the measurement was performed 60–90 min after the start of filtration for 120 min), while in [20], the permeance was evaluated within 120 min after the start of the experiment. Also, the membrane developed in this work exceeded the permeance value of the membrane obtained by us earlier [36] by 1.4 times, which was 95.7 L/(m^2^·h·bar). This phenomenon is due to a decrease in the molecular weight of the PPSU from 102 to 81 kg/mol and, as a consequence, the formation of a less dense porous structure that created hydraulic resistance to water flow. Thus, varying the molecular weight of the polymer makes it possible to create more highly permeable hollow fiber membranes from PPSU.

## 4. Conclusions

The influences of the molecular weight and chemical structure of polyphenylene sulfone (PPSU) end groups on the formation of the porous structure in ultrafiltration hollow fiber membranes were investigated. Polymers with a molecular weight in the range of 67–81 kg/mol and different ratios of hydroxyl and chlorine end groups were synthesized. According to NMR spectroscopy data of the synthesized PPSUs, it was found that an excess of one of the monomers during the synthesis leads to the expected predominance of hydroxyl (excess DHBP) or chlorine (excess DCDPS) end groups. 

The coagulation values and rheological properties of dope solutions based on synthesized PPSUs were investigated. It was shown that the viscosity of dope solutions increases with increasing molecular weight. However, a new and unexpected result is that the stability of PPSU solutions is determined not by the molecular weight of the polymer, but by the chemical structure of its end groups. With the increasing proportion of chlorine groups, the stability of polymer solutions increases. This may be due to the formation of donor–acceptor hydrogen bonds between macromolecules, which leads to an increase in the cohesion energy of polymer clubs. This promotes the coagulation of PPSUs and phase decomposition of the polymer solution.

Dope solutions with polyethylene glycol concentration close to the maximum PPSU/NMP/PEG = 20/50/30 wt.% were used for forming asymmetric hollow fiber membranes from the PPSU. Using the SEM method, it was shown that the chemical structure of the end groups in the synthesized PPSUs in the presented range of molecular weights had a determining influence on the porous structure of the membranes. An increasing molar fraction of end chlorine groups resulted in a more open porous structure of the outer surface of the hollow fiber membranes. This may be due to the greater stability of PPSU solutions with chlorine end groups, whose phase decomposition produces a more loose and permeable porous structure.

The maximum water permeance of UF hollow fiber membranes was achieved with the PPSU sample that had the highest content of chlorine end groups, amounting to 136 L/(m^2^·h·bar), demonstrating a high rejection of the model substance Blue Dextran (at 94.7%). Currently, this is the best result among hollow fiber membranes made of unmodified PPSU.

## Figures and Tables

**Table 1:** Monomer ratios during the synthesis of PPSUs.

Sample	DHBP:DCDPS
PPSU-1	1:1.02
PPSU-2	1:1
PPSU-3	1.005:1
PPSU-4	1.008:1

**Table 2:** PPSU solution compositions.

Sample	*C_p_*, wt.%	*C_solv_*, wt.%	Pore-Forming Agent	*C_por_*, wt.%
PPSU-1	20	80	-	-
PPSU-2	20	80		
PPSU-3	20	80		
PPSU-4	20	80		
PPSU-1	20	50	PEG	30
PPSU-2	20	50		30
PPSU-3	20	50		30
PPSU-4	20	50		30

**Table 3:** Results of the investigation of synthesized PPSUs using GPC and NMR methods.

Sample	M_p_, kg/mol	M_w_, kg/mol	M_n_, kg/mol	M_w_/M_n_	M_NMR_	DHBP:DCDPS in Synthesis	OH:Cl in Polymers	y_-OH_	y_-Cl_
PPSU-1	75	81	24	3.4	12.7	1:1.02	0.43	0.010	0.023
PPSU-2	56	67	25	2.6	10.5	1:1	1.7	0.020	0.012
PPSU-3	76	78	33	2.4	15.3	1.005:1	11	0.022	0.002
PPSU-4	64	72	44	1.6	15.7	1.008:1	17	0.017	0.001

**Table 4:** Thermal properties of the synthesized PPSUs.

Sample	Glass Transition Temperature T_g_, °C
PPSU-1	227.0
PPSU-2	225.9
PPSU-3	226.2
PPSU-4	228.5

**Table 5:** Filtration characteristics of hollow fiber PPSU membranes.

*M_w_*, kg/mol	*P*, L/(m^2^·h·bar)	Calibrant	*R*, %	Ref.
50	65	BSA ^1^	95.2	[20]
102	95.7	Blue Dextran ^2^	99.9	[36]
81	136		96	This work

^1^ Bovine serum albumin, M_w_ = 69 kg/mol. ^2^ Blue Dextran, M_w_ = 70 kg/mol.

## Data Availability

Data are contained within the article.
